# Automatic Classification and Visualization of Text Data on Rare Diseases

**DOI:** 10.3390/jpm14050545

**Published:** 2024-05-20

**Authors:** Luis Rei, Joao Pita Costa, Tanja Zdolšek Draksler

**Affiliations:** 1 Jožef Stefan Institute (IJS), Jamova 39, 1000 Ljubljana, Slovenia; luis.rei@ijs.si (L.R.); tanja.zdolsek@ijs.si (T.Z.D.); 2 Jožef Stefan Institute, Jožef Stefan International Postgraduate School, Jamova 39, 1000 Ljubljana, Slovenia; 3 International Research Centre for Artificial Intelligence under the Auspices of UNESCO (IRCAI), Jamova 39, 1000 Ljubljana, Slovenia; 4 Quintelligence, Teslova 30, 1000 Ljubljana, Slovenia; 5 IDefine Europe, Jamova 39, 1000 Ljubljana, Slovenia

**Keywords:** artificial intelligence, semantic technologies, rare diseases, healthcare, text mining, MeSH headings, MEDLINE mining, worldwide news

## Abstract

More than 7000 rare diseases affect over 400 million people, posing significant challenges for medical research and healthcare. The integration of precision medicine with artificial intelligence offers promising solutions. This work introduces a classifier developed to discern whether research and news articles pertain to rare or non-rare diseases. Our methodology involves extracting 709 rare disease MeSH terms from Mondo and MeSH to improve rare disease categorization. We evaluate our classifier on abstracts from PubMed/MEDLINE and an expert-annotated news dataset, which includes news articles on four selected rare neurodevelopmental disorders (NDDs)—considered the largest category of rare diseases—from a total of 16 analyzed. We achieved F1 scores of 85% for abstracts and 71% for news articles, demonstrating robustness across both datasets and highlighting the potential of integrating artificial intelligence and ontologies to improve disease classification. Although the results are promising, they also indicate the need for further refinement in managing data heterogeneity. Our classifier improves the identification and categorization of medical information, essential for advancing research, enhancing information access, influencing policy, and supporting personalized treatments. Future work will focus on expanding disease classification to distinguish between attributes such as infectious and hereditary diseases, addressing data heterogeneity, and incorporating multilingual capabilities.

## 1. Introduction

### 1.1. Motivation

Rare diseases affect a small number of people when considered individually. However, when considered collectively (within more than seven thousand rare diseases), they affect more than 400 million people worldwide and are spread across over ten thousand diseases [1]. Most rare diseases are neurodevelopmental disorders with characteristics that fit well the challenges of precision medicine [2]. Patients with rare diseases often face a number of difficulties, including misdiagnosis, and delayed diagnosis, the majority also have no treatment options [3]. Rare genetic diseases are at the core of personalized medicine, requiring precision diagnostics and precision therapeutics. Moreover, the methodology for precision medicine was developed within the rare disease community having broader implications for the rest of medicine [4], and so a perspective on the course of precision medicine goes for rare to common diseases. Associated with the problem of data and information coverage in this context, there is an opportunity for machine learning-based text mining technologies, particularly those that can overcome the language barrier in their information retrieval and interaction with the associated disease-driven communities worldwide.

### 1.2. Related Work

AI-based technologies are being accepted at a slow and cautious pace by the often-conservative health domain. The recent successes in the application of artificial intelligence (AI) in the digital transformation of other industries have helped progress in the development of new healthcare methods, particularly those requiring evidence in decision making. For rare diseases, there has been always the problem of small cohorts and a lack of available information, and with this paper, we show that text-mining-based technologies can help in that context. In recent years, we have been witnessing a positive change also in the domain of rare diseases, most often with machine learning (ML) technologies being used for disease diagnosis (usually imaging-based) (see [3,5,6,7]). In this area of knowledge, the number of biomedical publications and their heterogeneity create obstacles to its usage in meaningful ways.

When considering the more general classification of scientific articles with MeSH classes, the main references include MeSH Now [8], with its learning-to-rank framework; the MeSHLabeler [9], combining predictions from MeSH classifiers, KNN and pattern matching; and BERTMeSH [10], a pretrained deep contextual representation based on BERT, capturing deep semantics of full text. It is worth noting that since December 2021 the NLM, is transitioning to the automation of MeSH indexing assigning MEDLINE citations through the MEDLINE 2022 initiative [11].

Also related to this work, and specifically focusing on the annotation of news articles with MeSH terms, in 2021, we developed a text classifier learning the MEDLINE records labeled by MeSH Headings [12]. In this work, we build on the latter-mentioned work to improve queries of different rare disease topics in a wide range of health documents, from news articles to medical reports. The appropriate annotation of news is an important value of the proposed technology complementing the classification by Wikipedia terms used in the queries of the news engine Event Registry (ER) used in this study, able to collect and analyze over 300 thousand news articles daily [13] in more than 60 languages.

### 1.3. Contributions

This study describes a first approach to automatically categorizing texts based on disease attributes: particularly, whether or not a scientific or a news article is about rare or non-rare diseases. Our first contribution is the methodology used to create and evaluate a high-accuracy classifier for this task. This methodology couples the popular PubMed/MEDLINE archive with dedicated rare disease ontologies. Secondly, we publish our expert-annotated news dataset containing news articles labeled with MeSH terms, including the terms that refer to four rare NDDs. This dataset complements a pre-existing news dataset that contains articles related to non-rare diseases. The dataset provides a foundation for future research to validate and improve classification algorithms. Third, the rare diseases portal shows a live demonstration of the research results obtained, combining different global data sources, features, and visualizations that provide the appropriate information and knowledge to empower patients, researchers, and clinicians.

Finally, we provide a good foundation for future research. The outcomes of this study serve as a stepping stone for further explorations into disease classification. The methodology established here is easy to adapt to broader disease categorization contexts and expanded to accommodate multilingual text. Our analysis points towards data heterogeneity as one of the main future challenges. By providing the means for rare disease classification, this study contributes to better information accessibility. This has practical implications for shaping health policy, supporting personalized treatment strategies, and ultimately enhancing patient care for those suffering from rare diseases.

## 2. Materials and Methods

### 2.1. Preliminary Analysis of Rare Diseases in Research and the News

The purpose of this analysis is threefold: first, we need to establish some understanding of the data; second, we will have very limited expert annotation time, and we must make sure we select a viable pool of candidates, e.g., if the number of articles we can find about a rare disease is too small, it might be a bad candidate; thirdly, presenting this analysis provides data to substantiate the need for an automated text classifier to help identify articles related to rare diseases. We are aware of more than 7000 rare diseases, with around 5000 classified as neurodevelopmental disorders (NDDs), which are typical examples of rare diseases [14]. These NDDs often share overlapping features and clinical symptoms, allowing for collective analysis and study to a certain extent. For our preliminary analysis, we have chosen a set of 16, detailed in Table 1; most of these are monogenic disorders. This table highlights the underrepresentation of these disorders in both the scientific literature and news media. The disorders were chosen to represent a spectrum of familiarity, from lesser-known conditions like Kleefstra syndrome (formerly known as 9q34 deletion syndrome) to more recognized ones such as Angelman syndrome. Table 1 includes (i) the languages covered by Wikipedia, which indicates the multilingual reach when utilizing Wikipedia terms as per [13]; (ii) the number of news articles indexed by the ER news engine in 2022; (iii) the volume of scientific articles according to PubMed; and (iv) the year each corresponding MeSH term was introduced, shedding light on the historical data coverage in MEDLINE.

Fragile X syndrome has the largest presence in MEDLINE, with 7603 scientific articles, followed by Rett syndrome and the Prader–Willi syndrome, with 4381 and 4315 scientific articles, respectively. The MeSH categories were introduced with a scope of 46 years, between 1976 (Prader–Willi syndrome and Dravet syndrome) and 2012 (Kleefstra syndrome and Koolen–de Vries syndrome). Seven of these classes are main MeSH topics and nine are supplementary concepts. The corresponding language coverage for the corresponding Wikipedia concepts varies from concepts covered in 39 languages (Rett syndrome) to three languages (SYNGAP1 syndrome). The news coverage in 2022 shows a wide range of incidence from 1280 news articles (e.g., Rett syndrome) to 6 news articles (e.g., Koolen–de Vries syndrome). The expert annotation data for rare diseases are available at [15].

This preliminary analysis highlights significant disparities in the coverage of rare diseases across published science and online news articles. There are also notable differences in how news media, Wikipedia, and scientific articles address these conditions. Moreover, the year a disease was added to the MeSH database sheds light on the evolving definition of rare diseases, illustrating how our understanding has expanded over time. This evolution significantly impacts data coverage and collection

We will use four specific diseases in Section 2.3 to build our news article dataset: Angelman, De Lange, Fragile X, Kleefstra. The first three are within the rare diseases with more exposure, with the corresponding MeSH terms registered from 1982 to 1999. The last one, Kleefstra, was only registered in 1992 and is still a supplementary concept mapped to major terms such as *Chromosome Deletion* or *Intellectual Disability*. It will allow us to show the capabilities of the classifier discussed in this paper in the context of more challenging terms such as this.

### 2.2. Rare Disease Terms

MeSH is a comprehensive controlled vocabulary, developed and maintained by the National Library of Medicine (NLM) of the United States, used for indexing journal articles and books in biomedical sciences. It is an effective and consistent way to retrieve information that may utilize different terminology for the same concepts. It is used by PubMed a search engine for biomedical and life sciences literature, containing more than 36 million citations and abstracts, the largest part of which belongs to the MEDLINE dataset [16]. MEDLINE, a popular resource among biomedical researchers, includes over 30 million citations and abstracts from the biomedical literature, stretching back to 1966. Each year for the last ten years, approximately one million new articles have been added. The majority of entries are meticulously annotated by health professionals into 16 primary categories, with a detailed hierarchy that can extend up to 13 levels deep.

Although MeSH is comprehensive, it is not a perfect controlled vocabulary for all purposes and subjects. On the subject of diseases, numerous other controlled vocabularies exist, including OMIM, MedDRA, MedGen, Orphanet/ORDO, GARD, and Mondo. Some of these, such as ORDO and GARD, are specifically about rare diseases. MeSH contains an entire hierarchical tree for diseases (C); while MeSH itself includes a rare disease attribute term (D035583), it is neither a parent of all rare diseases nor used whenever a rare disease is the subject of a bibliographic entry. Even the dedicated rare disease databases need to answer the basic question of "How many rare diseases are there?". This question was the subject of a dedicated analysis conducted on the Mondo [17] semiautomatically constructed disease ontology [18]. Mondo includes a “rare” property assigned to all diseases that are classified in its ontology as being rare diseases. The general-purpose Wikidata knowledge base also includes a term for rare disease (Q929833). Mondo and GARD, for example, include links to the MeSH vocabulary. Upon conducting an exploration of these different ontologies, we selected Mondo because its “rare” property seemed to focus on diseases directly instead of including concepts (e.g., genes) potentially linked to them and because it has more potential for future work along other disease attributes such as hereditary vs. infectious diseases. Table 2 shows the term counts by ontology, for reference. We extracted all Mondo leaf terms that were marked as rare and had a direct link to MeSH or had a link to GARD, which in turn had the link to MeSH. This resulted in a list of 637 MeSH terms that refer to rare diseases. Through the MeSH tree, we added all children nodes of these and the MeSH rare disease term (D035583) for a total of 709 terms, with which we built our datasets in Section 2.3.

### 2.3. Dataset

We frame our problem as a multiclass text classification problem. This is a type of machine learning task where the goal is to predict which one of three or more discrete, mutually exclusive, categories (classes) a particular instance (data point) belongs to and where each instance corresponds to a text such as an abstract or a news article. The goal of this task is to learn parameters for a model of the data that generalizes from instance-label pairs, example samples, seen during a training phase, to predict the labels for unseen instances. During a testing phase, the label associated with the instance is withheld and used to evaluate the learned model. Thus, a dataset of example samples is required and must be divided into disjoint training and test subsets. It is further common to create another disjoint set of examples, called a validation set, that is used to determine parameters of the learning process, called hyperparameters, such as how many times the training examples are shown to the model during training.

To create our dataset, we utilize the PubMed data, which are publicly available for download. Most of their references include abstracts, from which we create our instances, and their corresponding MeSH headings, which are the basis for our labels. MEDLINE/PubMed data are an attractive source for creating machine learning datasets, a practice that started with the popular Ohsummed dataset [19] and was at the core of NewsMeSH [12]. Given the list of rare disease MeSH terms we obtained in Section 2.2 and the 2023 PubMed baseline data, we created a dataset of abstracts labeled to indicate whether they refer to rare diseases, non-rare diseases, or something else. We utilized a few simple heuristics to sample data from PubMed. We selected only journal articles with a date and a nonduplicate identifier, MeSH headings, a valid date, title, and abstract. Once joined, the title and abstract formed the text field of our records and, as a data quality selection heuristic, were required to contain at least 128 whitespace delimited tokens. Whitespace delimited tokens roughly correspond to words. This resulted in approximately 2M records. Each record was assigned to a category by the following method:

1. If it contained any MeSH heading in the list of 709 rare disease terms, it was assigned to the rare disease category (see Section 2.2);
2. Otherwise, if it contained any MeSH term in the Disease tree or the Mental Disorders (F03) tree, it was assigned to the non-rare disease category;
3. Otherwise, it was assigned to the “Other” category.

To facilitate experiments by reducing their computational cost, we randomly sampled 24,000 records for each class and randomly assigned each to a training, validation, or test set. The number of samples in each class is summarized in Table 3, with 60,000 examples allocated to the training set and 6000 examples each to the validation and test sets.

To evaluate our classifier on news data, we augmented the original NewsMeSH [12] dataset of 100 expert annotated news articles with an additional 40 evenly split between four different rare diseases: Angelman, De Lange, Fragile X, Kleefstra [15]. Each news article is expert-annotated with MeSH headings. We again utilized the list of MeSH terms from Section 2.2 to assign each news article to one of the three classes we defined for our dataset. Unlike the abstract dataset derived from PubMed/MEDLINE, which covers a broad range of rare diseases, our news article dataset includes only rare NDDs, as all selected diseases belong to this category. For instance, Granulomatosis with polyangiitis (GPA), although a rare disorder, is not an NDD, and is included in the abstract dataset but absent from our expert-annotated news dataset.

The number of samples for the resulting test set is detailed in Table 4. It is not viable to randomly sample news articles and expect a significant percentage of them to be about diseases, much less rare diseases. Thus, the sampling was not random and instead focused on including specific concepts. Thus, the percentage of articles that fall into the “Other” category is much smaller than it would be if the sampling was random. This is fine, since we expect a separate classifier such as NewsMeSH to have already categorized articles as being about diseases and expect only to further separate them into rare vs. non-rare.

### 2.4. Text Classification Model

**Figure 1:**
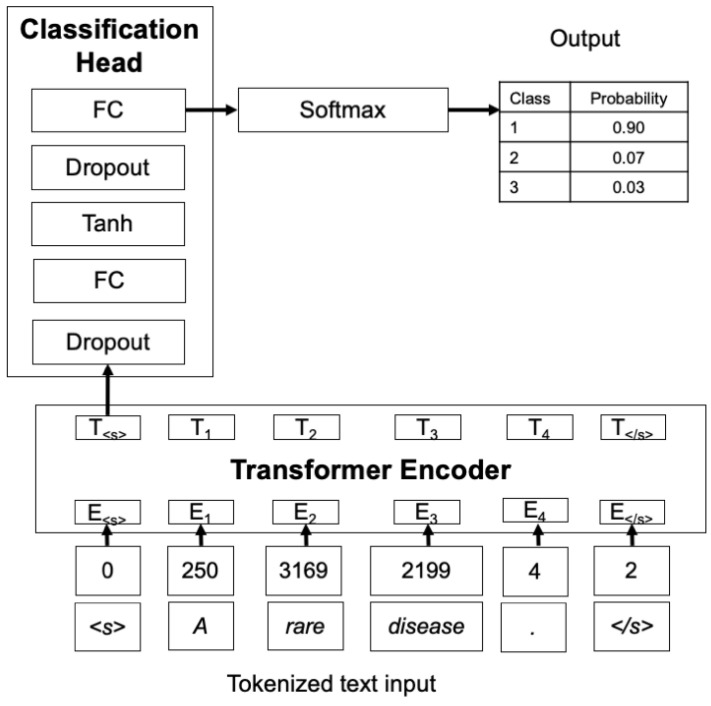
Architecture of the classifier model, where the transformer encoder and the classification head are the main blocks, in bold, and only the output of the start token will pass to the classifier.

Our text classifier is based on the architecture of BERT (bidirectional encoder representations from transformers) [20], which has become the de facto standard for developing text classifiers. BERT processes text by understanding the context of each word in relation to all other words in a sentence, rather than in isolation or sequence. This model encodes input text into a high-dimensional space, creating dense vector representations that capture both syntactic and semantic features crucial for classification. These vectors are then input into a classification head, typically comprising one or two fully connected neural network layers. During training, the weights of BERT’s language model and the classification head are jointly fine-tuned through backpropagation [21] on a supervised dataset, a process known as supervised fine tuning. BERT utilizes a stack of encoder layers that process the input sequence simultaneously using self-attention mechanisms. It also introduces a special classification token (CLS) at the beginning of each input sequence. The final hidden state of this CLS token serves as the aggregate sequence representation for classification tasks. The architecture operates within a fixed dimensionality, $d_{model}$, set to 768 in the BASE model, which defines the size of the hidden layers and the breadth of the model’s capacity to process and analyze text. We chose RoBERTa [22] as the encoder model and its publicly available weights. RoBERTa builds upon BERT but was trained on a larger dataset, had different hyperparameters, and removed the next-sentence pretraining objective. This model has a different tokenizer based on the GPT tokenizer [23], which breaks text down into subwords, allowing the model to handle rare words more effectively than traditional tokenizers. The CLS token of BERT is replaced by a sequence start token (“<s>”), which is functionally similar. The encoded text representation, which corresponds to the final hidden state of the start token, is then processed through a classification head, which derives the final output categories from the dense vector representations (the final hidden state of this CLS token). This head starts with a dropout layer [24], which helps prevent overfitting by randomly omitting a subset of features during training. Following the dropout, the sequence passes through a fully connected (dense) layer, activated by a hyperbolic tangent function. Another dropout layer follows, providing additional regularization before the final fully connected projection layer, which further refines the features for the output classification. The output of this layer is transformed by a softmax function into a probability distribution across the predefined classes, indicating the likelihood that the input text belongs to each category. The first fully connected layer has a dimension $d_{model}\times d_{model}$ and the projection layer has a dimension of $d_{model}\times K$, where *K* corresponds to the number of predefined categories or classes. In our case, K=3 (rare, non-rare, other). This classification head is the same as the Transformers library [25] in the “RobertaForSequenceClassification” model. We adopted it in this work to facilitate replication of our experiments. Figure 1 shows a schematic of the model.

### 2.5. Metrics

We will report experimental results using the standard performance metrics for classification problems: accuracy (Equation (1)), precision (Equation (2)), recall (Equation (3)), and F1-score (Equation (4)). Accuracy is determined by dividing the number of correct predictions, including both true positives and true negatives, by the overall number of instances. Precision for a specific class quantifies the fraction of accurately identified instances (true positives) within all predictions made for that class, including both correct (true positives) and incorrect predictions (false positives). Recall quantifies the proportion of instances accurately classified into a specific class relative to the total instances of that class, encompassing both correctly identified (true positives) and missed instances (false negatives). The F-score metric is computed as the weighted harmonic mean of precision and recall. Typically, the weight is set to 1, which equally emphasizes both precision and recall, resulting in the metric being referred to as the F1 score.

(1) $$\begin{array}{ccc} accuracy & = & \frac{TP+TN}{TP+TN+FP+FN} \end{array}$$

(2) $$\begin{array}{ccc} precision & = & \frac{TP}{TP+FP} \end{array}$$

(3) $$\begin{array}{ccc} recall & = & \frac{TP}{TP+FN} \end{array}$$

(4) $$\begin{array}{ccc} F_{1} & = & 2\frac{precision\cdot recall}{precision+recall} \end{array}$$

When multiple classes are present, there are two common techniques for averaging the metrics for each class. Micro-averaging aggregates individual true positives, false positives, and false negatives across all classes to calculate overall metrics. In contrast, macro-averaging computes metrics for each class independently and then averages them. Micro-averaging is sensitive to class imbalance, favoring larger classes, while macro-averaging gives equal weight to all classes. We will report both averages.

We define a background class, “Other”, to represent all those inputs that do not belong to any of the specific, targeted classes the model is trained to identify, namely, “Rare Diseases” and “Non-rare Diseases”. This class acts as a catch-all category for any data that is irrelevant. As is common practice, we omit correctly classified instances associated “Other” from the average scores and do not show its class-specific results since they are, by definition, irrelevant.

## 3. Results

We initialized our model weights with the 125M parameter pretrained ’RoBERTa-base’ weights. Following the procedure of the original RoBERTa evaluations [22] we trained our model using the Adam optimizer [26] for 10 epochs and selected the model at the end of the epoch with the best results. The metric for selecting the best model was the micro-averaged F1-score without the background class. The other hyperparameters, namely the batch size and the learning rate, were also selected from the RoBERTa paper. Table 5 shows the values of the hyperparameters the model was trained with.

**Figure 2:**
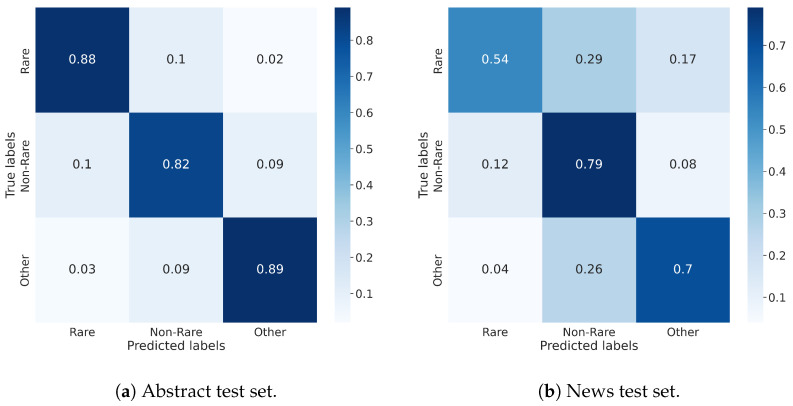
Confusion matrices.

Table 6 and Table 7 show the summary results on our dataset of PubMed abstracts and news, respectively. The corresponding accuracies are 0.86 and 0.70, respectively. Figure 2a,b show their respective confusion matrices. The results are very good for classification of journal abstracts, with around 85% average for all metrics. While less good, the results for news are still very good at around 70%. This is significantly higher than the results for assigning labels reported in NewsMeSH, although that is expected as that task is considerably more challenging. Results on the abstracts test set are significantly better than on the news test set. This is expected and due to the fact that the training set consisted only of abstracts, and news is a significantly different domain. Also expected is the fact that the primary confusion occurs between rare and non-rare diseases.

## 4. Community-Driven Exploration of Rare Diseases Data

**Figure 3:**
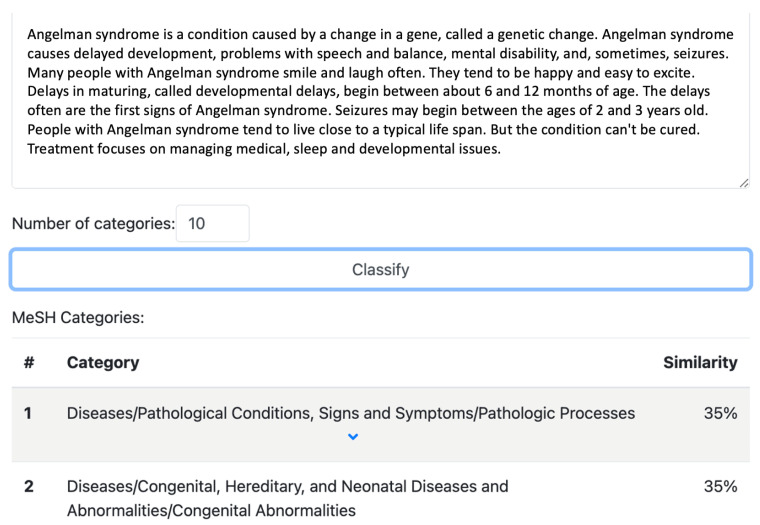
The MeSH classifier for health-related documents.

The proposed methodology and MeSH classifier in this paper can be implemented in any news engine as a new rare-diseases-specific classifier, to greatly improve the obtained results. It can also be used independently, for datasets of news media or any other text-based documents (e.g., medical reports), offering efficient automated annotation of the input text, assigning to it the MeSH categories and probability of text similarity (limited to English text-based input, taking into consideration that MEDLINE and MeSH only exist in English language). The online demonstrator of the MeSH classifier [27] displays both the rank of the MeSH term in the annotation and the term’s significance as a percentage, calculated using cosine similarity (see Figure 3). Additionally, this classifier can be accessed via a REST API that responds to POST requests with JSON input containing the text to be classified.

**Figure 4:**
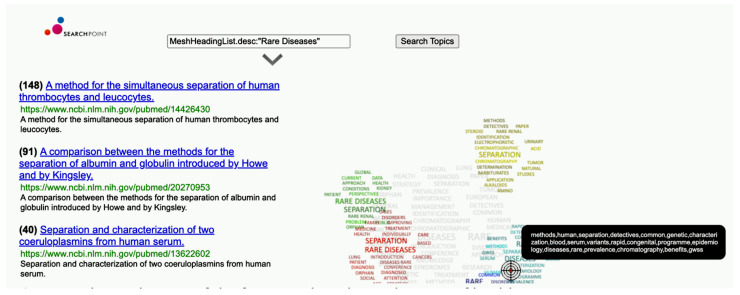
The application of the framework to the exploration of health news.

Considering the vast volume of publications and the challenges in accessing scientific information, we have provided a MEDLINE explorer, available at [27], where users can search the system and use a pointer to refine their search results (for instance, finding articles on biomarkers associated with “Rare Diseases” MeSH descriptors). To further aid in the exploration of texts related to rare diseases, including scientific reports and news, our automated classifier tags the input text with relevant MeSH classes, allowing for enhanced navigation through text queries using Lucene syntax. The complex visualization proposed in this framework for interactive MEDLINE data exploration, initially developed and validated in [12], uses text similarity for literature review, as represented in Figure 4, showing a scientific article originally positioned as 148th, now in 1st place due to its proximity to the specific topic. The integration of the MeSH classifier with this complex visualization system allows us to utilize MeSH categories as search terms in a query, leveraging the machine learning capabilities to support literature review.

**Figure 5:**
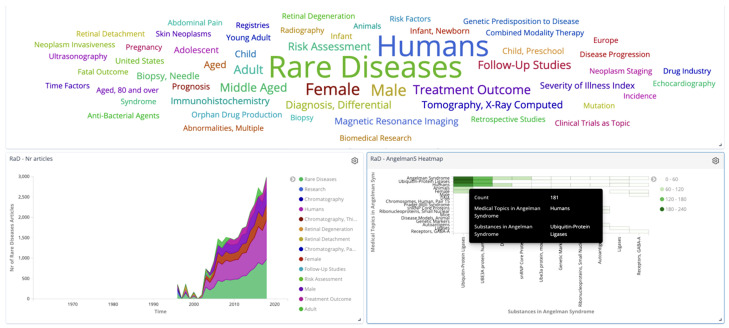
The data exploration tool allowing prototyping by health experts.

To facilitate the discussions with the parent-led associations and expert communities, and better understand what could be meaningful ML methods, analysis outputs and data visualizations, without the need for much technical expertise, we have developed a data visualization dashboard, providing the user with real-time access to the MEDLINE dataset. Its based on the Elasticsearch technology, using the Kibana open-source data visualization plugin (see Figure 5). With it, we allow for fast prototyping on previously prepared and pre-processed data samples, retracting rare diseases in general and specific syndromes in focus in this study. Part of this work with stakeholders initiated in the context of the European Union research project MIDAS [28], providing data-driven tools to support decision making, where one of the pilots was mental health [29]. By applying the know-how obtained in building a knowledge extraction and exploration system based on MEDLINE, we take into consideration the invaluable input on the usage of this prototype tool to discuss the meaningfulness of further research and development with the application of machine learning methods to the analysis of rare disease data. The input data enriched by the automated classifier feeds the index in Elasticsearch where the MEDLINE data and metadata are available. This new input generates changes in the data visualization modules that compose the rare diseases dedicated dashboard based on Kibana and the corresponding public instance. The most relevant views of this exploratory system allow the user to explore the ingested data and associated metadata, save the data sample queried to build predefined data visualization modules from templates and compose the latter into topic-focused dashboards populated with interactive charts and heat maps that can provide insight to the user and be shared as public instances.

## 5. Discussion

In this work, we showed our methodology for creating an accurate classifier that can label both research articles and news articles as being about either rare diseases or non-rare diseases. This is meant as an addition to a classifier that can label articles with MeSH terms, such as NewsMeSH. We overcame the limitations of MeSH regarding the classification of diseases by leveraging other ontologies, namely Mondo, although others such as ORDO, GARD, and Wikidata contain similar information. It is likely that this proposed methodology can be extended to classify text about diseases according to other categories, such as whether they are about infectious or hereditary diseases, as this information is also present in ontologies such as Mondo and Wikidata.

The ingested documents can be of all sorts, from electronic health records to medical reports, although as we have seen, classification performance drops when classifying news articles. This suggests that future work in domain adaptation or generalization is likely to be impactful. Another interesting avenue for future work is to extend it beyond English language texts, multilingual approaches as seen in [30] look promising. This methodology was developed in the context of information retrieval within online portals, dashboards, and observatories that can facilitate access to information about a specific topic, primarily for researchers, professionals, and policymakers. In this case, the topic falls within a category which we broadly call public health intelligence and more specifically, rare diseases. This in the sense that the there seems not to be a common agreement on the definition, taking into consideration the way the MeSH categories related to some of these diseases are only supplementary concepts and not main terms, as discussed in Section 2. The approach in this paper can also facilitate the development of a real-time recommendation system focusing on the challenges in rare diseases, with a significant amount of historical data about target diseases ingested from publications and news, health records and guidelines, but also crowd-sourced by patient communities and related nongovernmental organizations. We are exploring the potential of a rare disease-focused text-mining-based recommendation system that can be valuable in healthcare decision making and support access to quality information by parent-led associations, promoting more efficient care in these often overlooked areas.

We need to highlight that there were several limitations in this study beyond those discussed in the context of future work. The first is that, at present, there is no complete and universally agreed list of rare diseases. We took the Mondo ontology and its MeSH tree children, for a total of 709 terms, as our list, but pointed out that other ontologies would result in different lists of rare diseases with significantly different numbers of MeSH terms. MeSH itself is not instantly updated and the PubMed/MEDLINE articles using it may not have been associated with the term, often because it might not have existed at the time of cataloging. The second major limitation of this study is the attention given to rare NDDs. Although the larger abstract dataset, part of which was used to train the classifier, includes a broader set of rare diseases, only monogenetic NDDs were selected for inclusion in the news dataset and analyzed in Table 1. This selection bias is partially justified because this group, representing approximately 5000 of over 7000 rare diseases, shares overlapping features and clinical symptoms, making it feasible to study them collectively. This approach is practical for a pilot study; however, future expansions of the news dataset should aim to encompass a broader range of rare diseases. The third major limitation of our study is the small size of our news dataset, which contains only 140 articles, with just 40 covering rare diseases. Creating manually annotated datasets is both costly and time-consuming, especially when annotations require healthcare experts familiar with MeSH terms. While semiautomatic tools like the NewsMeSH classifier and its user interface [12] can mitigate some of these challenges, they do not eliminate them. We augmented an existing dataset to build our current dataset and anticipate that future efforts will continue to expand upon this work.

## Figures and Tables

**Table 1:** Coverage of the 16 selected rare diseases over MEDLINE and international news media, indicating the language coverage of the corresponding Wikipedia concept, the number of news articles in the year 2022, the number of scientific articles until 2020, and the year of the corresponding MeSH concept.

Rare Disease	Languages	News Articles	Scientific Articles	MeSH Year
Kleefstra syndrome	9	105	127	2012
Angelman syndrome	35	487	2036	1992
Dravet syndrome	18	676	1555	1976
Cornelia de Lange syndrome	19	136	855	1999
Phelan–McDermid syndrome	14	55	337	2010
Fragile X syndrome	32	1091	7603	1982
Pitt–Hopkins syndrome	11	92	180	2010
Prader–Willi syndrome	38	1296	4315	1976
FOXG1 syndrome	4	22	51	1994
Koolen–de Vries syndrome	5	6	50	2012
Wiedemann–Steiner syndrome	5	10	79	2009
Kabuki syndrome	14	67	592	2010
Rett syndrome	39	1280	4381	1989
SYNGAP1 syndrome	3	75	63	2004
SATB2 syndrome	5	79	92	2007
CTNNB1 syndrome	14	289	3	2005

**Table 2:** Number of rare disease MeSH term counts in each ontology. Mondo, in bold, was selected for this work.

Ontology	MeSH Terms
GARD	1265
ORDO	1052
**Mondo**	637
Wikidata	476

**Table 3:** Number of samples in our PubMed dataset split per set and per class.

Subset	Samples per Class	Total
Training	20.000	60.000
Validation	2.000	6.000
Test	2.000	6.000
Total	24.000	72.000

**Table 4:** Number of samples in our news article test set.

Class	Samples
Rare Diseases	41
Non-Rare Diseases	72
Other	27
Total	140

**Table 5:** Hyperparameter values used for training the supervised model on our dataset.

Hyperparameter	Value
Batch Size	32
Learning Rate	$3\times 10^{-5}$
Max Epochs	10

**Table 6:** Results on the abstract test set.

Class	Precision	Recall	F1
Rare Diseases	0.88	0.88	0.88
Non-rare Diseases	0.82	0.82	0.82
Other	0.89	0.89	0.89
Averages			
Micro	0.86	0.86	0.86
Macro	0.86	0.86	0.86
Averages without “Other”			
Micro	0.85	0.85	0.85
Macro	0.85	0.85	0.85

**Table 7:** Results on the news test set.

Class	Precision	Recall	F1
Rare Diseases	0.69	0.54	0.60
Non-rare Diseases	0.75	0.79	0.77
Other	0.59	0.70	0.64
Averages			
Micro	0.70	0.70	0.70
Macro	0.68	0.68	0.67
Averages without “Other”			
Micro	0.73	0.70	0.71
Macro	0.72	0.66	0.68

## Data Availability

The manually annotated news data are freely available online at [15].

## Abbreviations

The following abbreviations are present in this manuscript:

AI	Artificial Intelligence
ML	Machine Learning
NDDs	Neurodevelopmental Disorders
